# The Protective Effects of Icariin against the Homocysteine-Induced Neurotoxicity in the Primary Embryonic Cultures of Rat Cortical Neurons

**DOI:** 10.3390/molecules21111557

**Published:** 2016-11-22

**Authors:** Xiao-Ang Li, Yuen-Shan HO, Lei Chen, W.L. Wendy Hsiao

**Affiliations:** 1 State Key Laboratory of Quality Research in Chinese Medicine, Macau Institute for Applied Research in Medicine and Health, Macau University of Science and Technology, Av. Wai Long, Taipa, Macau, China; 1309853pct20001@student.must.edu.mo; 2 School of Nursing, The Hong Kong Polytechnic University, Kowloon, Hong Kong, China; janice.ys.ho@polyu.edu.hk; 3 Department of Genetics, Rutgers University, New Brunswick, NJ 07001, USA; larichen7@gmail.com

**Keywords:** homocysteine, neuroprotection, icariin, RT^2^ Profiler PCR array

## Abstract

Icariin, an ingredient in the medicinal herb *Epimedium brevicornum* Maxim (*EbM*), has been considered as a potential therapeutic agent for neurodegenerative diseases such as Alzheimer’s disease (AD). Hyperhomocysteinaemia is a risk factor for AD and other associated neurological diseases. In this study we aim to investigate whether icariin can reverse homocysteine (Hcy)-induced neurotoxicity in primary embryonic cultures of rat cortical neurons. Our findings demonstrated that icariin might be able restore the cytoskeleton network damaged by Hcy through the modulation of acetyl-α-tubulin, tyrosinated-α-tubulin, and phosphorylation of the tubulin-binding protein Tau. In addition, icariin downregulated p-extracellular signal-regulated kinase (ERK) which is a kinase targeting tau protein. Furthermore, icariin effectively restored the neuroprotective protein p-Akt that was downregulated by Hcy. We also applied RT^2^ Profiler PCR Arrays focused on genes related to AD and neurotoxicity to examine genes differentially altered by Hcy or icariin. Among the altered genes from the arrays, ADAM9 was downregulated 15 folds in cells treated with Hcy, but markedly restored by icariin. ADAM family, encoded α-secreatase, plays a protective role in AD. Overall, our findings demonstrated that icariin exhibits a strong neuroprotective function and have potential for future development for drug treating neurological disorders, such as AD.

## 1. Introduction

Homocysteine is a sulfur-containing amino-acid involved in the metabolism of cysteine and methionine. Level of Hcy is regulated by the remethylation and transsulfuration pathways [1]. The plasma level of total Hcy is between 5 to 15 μmol/L under the normal condition [2]. Hyperhomocysteinaemia, characterized by an abnormally high level of Hcy, can be caused by many factors [3]. Previous reports have shown that hyperhomocysteinaemia is a risk factor for vascular diseases such as stroke [4] and myocardial infarction [5,6]. It has also found to be associated with neurological disorders, including AD [7,8,9,10], Parkinson’s disease (PD) [11] and elderly depression [12,13]. Results from a prospective study showed that mild to moderate elevation of Hcy level increases the risk of AD. A 5 μmol/L increment of Hcy level increases the risk of AD by 40% [10]. The underlying mechanisms of Hcy toxicity remain unknown, but there is evidence from cellular and animal experiments showed a high level of Hcy can induce toxicity to different types of tissues [14,15,16]. Despite the different causes of hyperhomocysteinaemia, Hcy-lowering therapy is mostly confined to nutritional supplementations with vitamins B6, B12 and folic acid [17,18]. However, their effectiveness to alleviate the Hcy-related medical conditions, especially for neurodegeneration is still in doubt [19].

**Figure 1:**
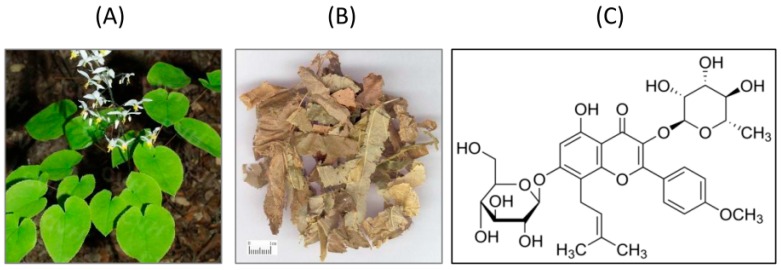
Photos of *Epimedium brevicornum* Maxim (**A**) The plant (**B**) The dry leaves (**C**) The chemical structure of icariin.

Icariin is present in all species of Epimedium herbs and is an indicative constituent of the Epimedium genus. In general, the contents of icariin in different Epimedium species varied from 0.003% to 1.55% using RP-HPLC analysis, or it can reach 3.69% in certain species [20]. Icariin (C_33_H_40_O_15_, 2-(4’-methoxylphenyl)-3-rhamnosido-5-hydroxyl-7-glucosido-8-(3’-methyl-2-butyleny)-4-chromanone, Figure 1) is a flavonoid extracted from traditional Chinese medicinal plant *Epimedium brevicornum* Maxim (Herba Epimedii; family Berberidacae; Ying-Yang-Huo in Chinese). It exerts a broad range of pharmacological and biological properties, including estrogenic activity, anti-inflammatory, antioxidant and anti-tumor effects, as well as cardio and neuronal protective effects [21,22,23,24]. In addition, icariin also showed effect on the memory deficits in aluminum-treated rats [25]. Similar studies have demonstrated that orally feeding of icariin can improve spatial learning ability and memory in both healthy and AD rats, suggesting that icariin can cross blood-brain barrier and modulate the brain functions [26,27]. The early pharmacokinetic study has revealed the presence of icariin and its major metabolites icarisides I & II in the rat plasma upon intragastric feeding the rats with icariin [28].

In this study we aim to investigate whether icariin can reverse the low dosage Hcy-induced neurotoxicity in primary embryonic culture of rat cortical neurons and the underlying mechanism.

## 2. Results 

### 2.1. Icariin Protected Primary Embryonic Rat Cortical Neurons from Hcy-Induced Cytotoxicity

**Figure 2:**
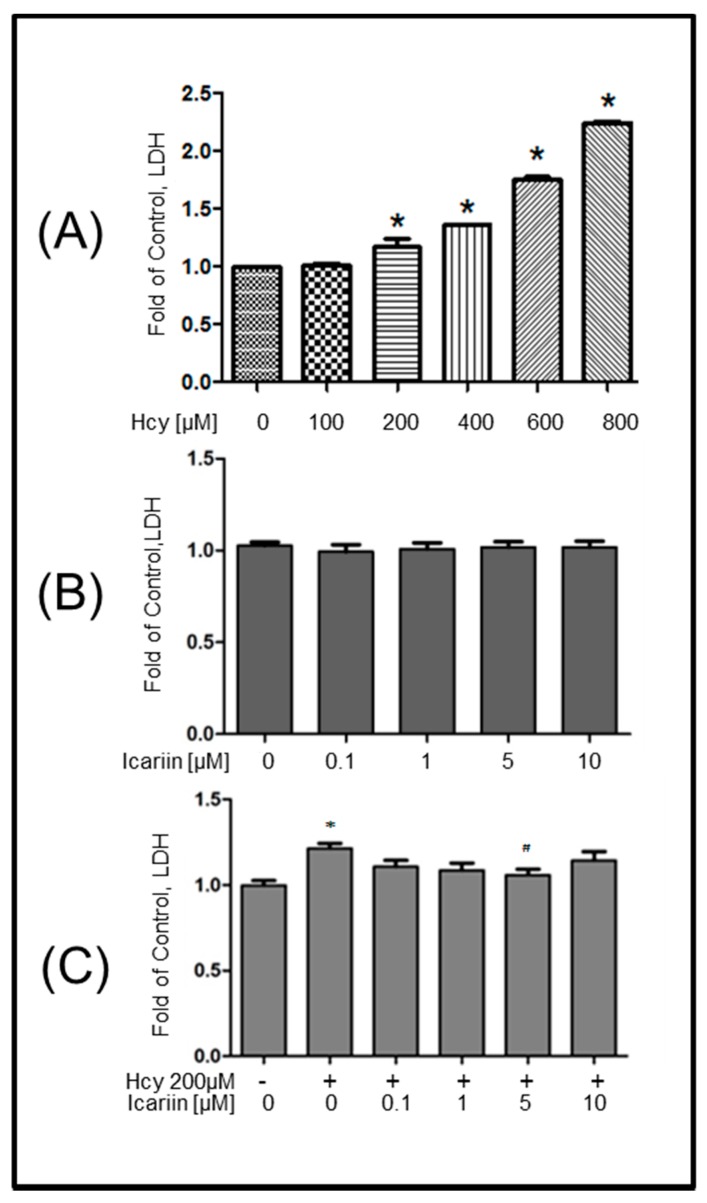
Cytotoxicity of Hcy and icariin measured by LDH assay. Cortical neurons were treated with Hcy (**A**), Icariin (**B**) and Hcy plus icariin (**C**) and performed LDH assay. The treatment duration for Hcy or Icariin was 12 h. In the co-treatment, cells were pretreated 1 h before the addition of Hcy. Results were quantified and expressed as fold of control. Data represents mean ± SE from at least three independent experiments. The significance of differences among treatment groups was determined by one-way ANOVA. *: *p* < 0.05 compared to control. “#”: *p* < 0.05. “+” and “-”: with and without the compound.

Elevated serum levels of Hcy can result in neurotoxicity. To better understand the effect of moderate dosage of Hcy as a risk factor for the chronic degeneration of the brain, we first exposed the primary embryonic culture of rat cortical neurons to an increasing concentration of Hcy (100, 200, 400, 600, 800 μM) for 12 h. As shown in Figure 2A, neuronal cell injury of Hcy-treated cells was examined by measuring the release of LDH in the culture medium. The results were expressed as fold of control. Hcy did not induce LDH on neurons at 100 μM, but it significantly increased the release of LDH from neuronal cultures at 200 μM and higher concentrations in a dose-dependent manner. The cytotoxic effect of icariin was examined at the dosages of 0.1, 1, 5, 10 μM for 12 h, and no cell injury was found (Figure 2B). In order to investigate the possible neuroprotective effects of icariin on Hcy-induced cell damage, neuronal cells were pretreated with icariin at non-toxic dosages of 0.1, 1, 5 and 10 μM for 1 h, followed by the co-incubation with Hcy (200 μM) for 12 h. As shown in Figure 2C, the viability of the culture exposed to Hcy was reduced, compared with the vehicle-treated control. Pre-treatment with icariin significantly reduced Hcy-induced cell injury in a dose-dependent manner, with a maximal effect obtained at 5 μM.

### 2.2. Icariin Restored the Hcy-Induced Acetylation and Phosphorylation of Tubulin in the Cultured Neuronal Cells

**Figure 3:**
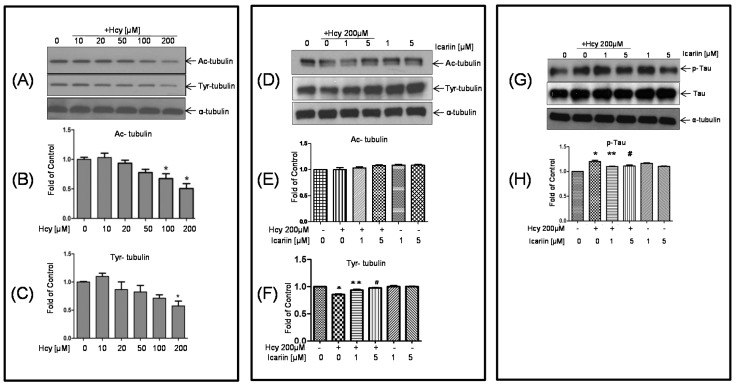
Icariin restored the Hcy-reduced acetylation and phosphorylation of tubulin in cultured neuronal cells. (**A**–**F**): Primary cortical neuronal cultures were treated with 200 µM Hcy for 48 h. For the co-treatment, cells were pretreated with icariin for 1 h, before incubating with Hcy for 48 h. The treated cells were subjected for western blot analyses and quantified for the detection of Ac- and Tyr-tubulin with anti-Ac-α-tubulin and anti-tyr-antibodies. (**G**–**H**) Western blot analysis for the detection of p-tau and total tau with anti-p-tau and anti-tau antibodies. Cells were treated 200αM Hcy alone for 2 h. For the co-treatment, cells were pretreated with icariin for 1 h, followed by co-incubation with Hcy for another 2 h. α-tubulin was used as internal control. Data represent mean ± SE from at least three independent experiments and was quantified using densitometry. *: *p* < 0.05 compared to control. ** and #: *p* < 0.05 compared to cultures treated with 200 μM Hcy. “+” and “-”: with and without the compound.

Acetylation and tyrosination play a role in the stability of tubulin and microtubules assembly [29]. To investigate the cytotoxic effect of Hcy on tubulin, cultured rat cortical neurons were treated with 10, 20, 50, 100, and 200 μM Hcy for 48 h, and examined the levels of acetylated-α-tubulin (Ac-tubulin) and tyrosinated-α-tubulin (Tyr-tubulin). As shown in Figure 3A–C, western blotting analysis revealed that the intensity of both Ac- and Tyr-tubulin decreased as the dosages of Hcy increased. Significant decrease in the intensity of Ac-tubulin at the dosages of 100 and 200 μM. Hcy at 200 μM also markedly decreased the intensity of Tyr-tubulin. Pretreatment with icariin at 1 and 5 μM for 1 h, followed by the co-incubation with Hcy (200 μM) for 48 h, Ac- and Tyr-tubulin were measured using western-blot analysis. The reduction of Tyr-tubulin, was restored to the level of the untreated mice with 1 and 5 μM icariin. Hcy treatment only showed mild reduction of Ac-tubulin, co-treatment with icariin restored or even enhanced the level of Ac-tubulin (Figure 3D–F).

### 2.3. Icariin Decreased the Level of p-Tau Which Was Deregulated by Hcy Treatment

Tau is a heterogeneous microtubule-associated protein that promotes and stabilizes microtubule assembly, especially in the axons. Tau is hyperphosphorylated at approximately 25 sites by ERK, GSK-3, and CDK5. Phosphorylation decreases the ability of tau to bind to microtubules and leads to instability of the microtubules. To investigate the status of tau phosphorylation, neuronal cells were treated with Hcy alone, or co-incubated with icariin for 2 h. For the co-treatment, cells were preincubated with icariin before the addition of Hcy. In Figure 3G,H, using the anti-p-tau antibody specific for the sites of Ser400/Thr403/Ser404, we observed that the level of p-tau increased after 1 h of Hcy incubation and reached to a peak at 2 h. Icariin at 1 and 5 μM significantly reduced the level of p-Tau in the treated neuronal cultures.

### 2.4. Immunohistochemical (IHC) Staining of Tubulin and Tau Proteins in Cortical Neuronal Cultures under the Treatments of Hcy and Icariin

**Figure 4:**
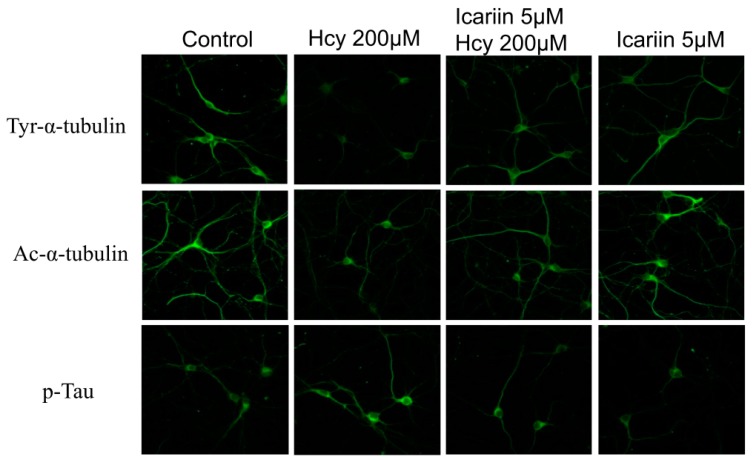
Microscopic images of IHC stained cells hybridized with anti-Ac-α-tubulin, anti-tyr-α-tubulin, and anti-p-tau (Ser400/Thr403/Ser404) antibodies. Method for IHC staining was described under Materials and Methods.

To further investigate the status of tubulin and tau protein in situ, the rat cortical neuron cultures were pre-treated with 5 μM icariin for 1 h, followed by a challenge with 200 μM Hcy for 48 h. The treated cells were subjected to IHC staining with Ac-tubulin, Tyr-tubulin, or p-Tau antibody. Results showed (Figure 4) that Ac-tubulin and Tyr-tubulin fluorescence signals were decreased in Hcy-treated cells compared to the icariin and the control groups. In the case of p-Tau, the fluorescence signal in the Hcy-treated cells was stronger than the rest of the three groups. These results further suggested that icariin effectively stabilized cytoskeleton in the cortical neurons by increasing the tyrosination and acetylation of tubulin, while reducing the phosphorylation of tau.

### 2.5. Icariin Counteracts the Effects of Hcy on ERK, and AKT Signaling Molecules

According to our and others’ findings, Hcy can induce phosphorylation of tau, a possible link to the development of AD [30,31,32]. Tau can be phosphorylated by the mitogen-activated protein (MAP) kinases including ERK and c-Jun N-terminal kinases (JNK) [33,34,35].

**Figure 5:**
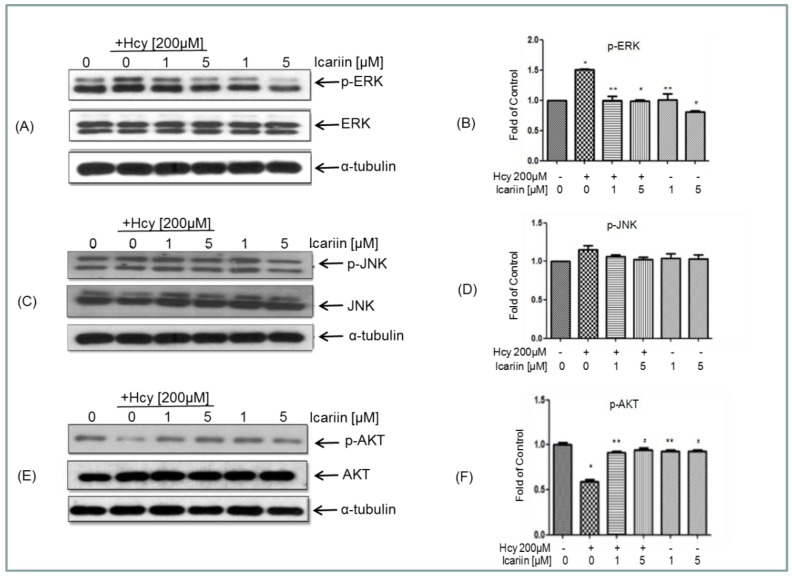
Icariin counteracts the effects of Hcy on ERK (**A**,**B**), JNK (**C**,**D**) and AKT (**E**,**F**) signaling molecules assessed using the western blot analysis. Cells were treated with either Hcy, Icariin, or both according to the treatment illustrated in Figure 3 legend. The α-tubulin was used as internal control. Statistical analysis was performed with one-way ANOVA. *: *p* < 0.05 compared to control. # and **: *p* < 0.05 compared to cultures treated with Hcy (200 μM). Data represent mean ± SE from at least 3 independent experiments. “+” and “-”: with and without the compound.

We investigated any changes of these kinases after Hcy treatment and found that the level of p-ERK increased after 1 h of Hcy incubation and reached to the peak at 2 h of incubation. Icariin at 1 or 5 μM significantly reduced the level of p-ERK in neuronal cells (Figure 5A,B). The level of p-JNK was also examined. There is a trend of increased level of p-JNK at 2 h. Neuronal cells treated with icariin showed reduction in the level of p-JNK, but not statistically significant (Figure 5C,D). These results indicate that ERK, instead of JNK might be involved in the protective effect of icariin. Previous study has shown that the activation of Akt pathway can protect neuronal cells from Hcy-induced apoptosis [36]. We investigated if Hcy could cause alteration in Akt phosphorylation in our cell model. As shown in Figure 5E,F, incubation with Hcy reduced the level of p-Akt. This reduction of phosphorylation of Akt was markedly restored by pretreatment with 1 and 5 μM icariin.

### 2.6. Using RT^2^ Profiler PCR Array to Investigate the Expression Profiles in Neuronal Cells under Icariin/Hcy Treatments

In addition to the known signaling molecules associated with the neuroprotective effects of icariin, in order to understand the action of icariin further, we applied RT^2^ Profiler PCR Arrays to look for AD- and neurotoxicity-focused genes expressed in four groups of neuronal cultures, i.e., (1) Hcy; (2) Hcy & icariin; (3) icariin; and (4) no treatment control. The AD array covered 84 genes important in the onset, development, and progression of AD; and the neurotoxicity array covered 84 key genes involved in drug and chemical-induced neurotoxic responses in neurotoxicity.

**Figure 6:**
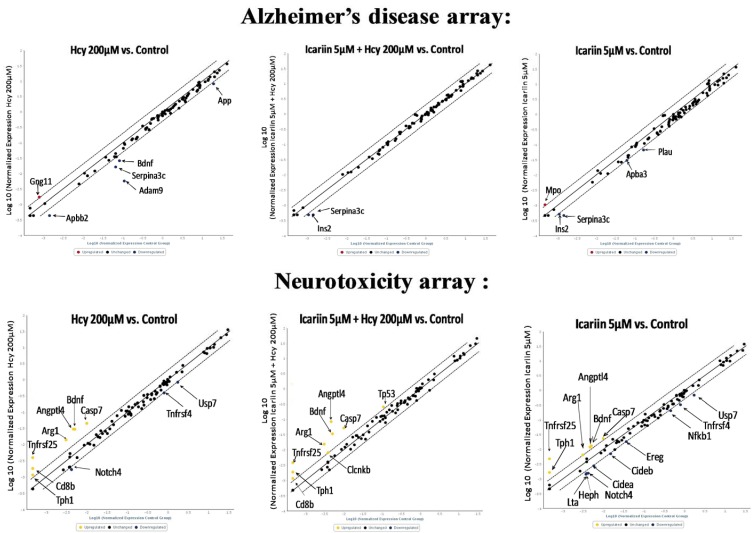
The scatter plots of AD array and the neurotoxicity array. cDNAs were prepared from Hcy, icariin, Icariin + Hcy and the control groups and each cDNA sample was applied for AD and neurotoxicity arrays. The scatter plots of each treatment groups were generated by normalized against the control according the Qiagen online data analysis for RT^2^ Profile PCR Arrays.

The results are represented by scatter plot (Figure 6). Eight genes from the AD array (Table 1) and eighteen genes from the neurotoxicity array were altered compared with the control.

**Figure 7:**
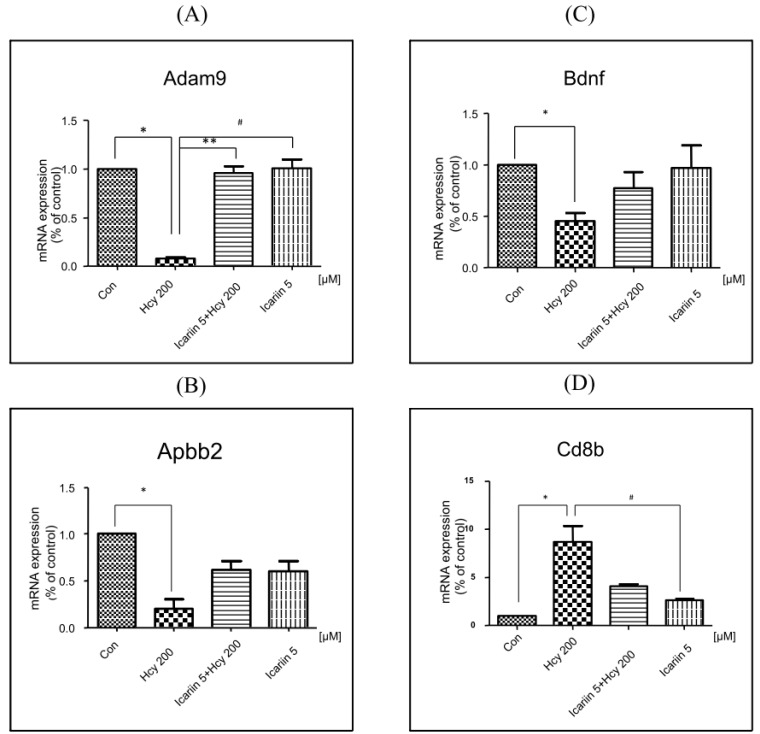
Verification of the mRNA expressions of four differentially altered genes identified from the AD and neurotoxicity arrays by using qPCR reactions with each specific primers. The mRNA levels of Adam9 (**A**), Apbb2 (**B**), Bdnf (**C**) and Cd8b (**D**) were verified using real-time RT-PCR with SYBR Green Mater and specific sets of primers for each gene (Table 1). All data was expressed in fold changes compared to the control. The house-keeping genes were used as internal control. Statistical analysis was performed with one-way ANOVA. *: *p* < 0.05 compared to control. ** and #: *p* < 0.05 compared to cultures treated with 200 μM Hcy.

Four genes that displayed differential expressions in Hcy- and icariin-treated cells and verified for their expressions using qPCR reactions with each specific primers (Table A1). These four genes are ADAM metallopeptidase domain 9 (Adam9), Amyloid β (A4) precursor protein-binding, family B, member 2 (Apbb2), Brain-derived neurotrophic factor (BDNF) and CD8b molecule (Cd8b). The results are shown in Figure 7.

BDNF is an important factor in neuronal survival. The expression of BDNF obtained from two sets of arrays (i.e., the neurotoxicity and the AD arrays) yielded contradictory results. However, real time RT-PCR with BDNF specific primers confirmed that mRNA expression of BDNF was downregulated by Hcy, and restored by icariin.

## 3. Discussion

The pathological changes of AD always start much earlier than the onset of disease symptoms [37]. Therefore, it has been proposed that early intervention on the underlying pathology is the key to successful AD therapies [38]. Recent translational research emphasizes on disease-modifying agents to target the early events of AD. Hyperhomocysteinaemia has been considered as one of early symptoms associated with a number of neurological disorders including AD [39,40,41]. To build an experimental model to mimic the early onset of AD where the degree of neuronal injury is low, we chose low cytotoxic dosage of Hcy in the primary embryonic cortical neuronal cultures throughout the study. Massive neuronal cell loss is only observed in late stage of AD. A lower dosage of Hcy which induces progressive degenerative changes is more relevant to the disease pathogenesis. We aware that the dosages we used in testing the protective effectives of Icarrin is still higher than the one observed in human subjects with severe hyperhomocysteinaemia. However, this is common as we used a cell culture model, in which other potential systematic response to icarrin that can affect the brain had been eliminated. In this study, using Hcy-induced neurotoxicity model in primary embryonic cortical neuronal cultures, we explored the potential of icariin as a modifier treating early onset of neurological disorders, including AD. Our findings showed that treatment with 200 µM of Hcy induced clear damage to the cortical neurons evidenced by the increase of neuronal cell injury and the LDH release, decrease of Ac- and Tyr-tubulin in the Hcy-treated cells. Treatment with icariin significantly restored the levels of Ac-tubulin and Tyr-tubulin (Figure 2A–C). Ac-tubulin, along with Tyr-tubulin plays an important role in the stability of microtubules as well as enhances intracellular trafficking [29,42]. Recent evidence showed that inhibition of histone deacetylase and elevation of Ac-tubulin in cultured rat cortical neurons reduced the necroptosis of neurons during ischemia-reperfusion [43]. In addition to the modulation of tubulin, icariin also effectively suppressed the elevation of p-tau that was induced by Hcy (Figure 3D–H). Tau is a microtubule- associated protein. It binds and induced Ac-tubulin and Tyr-tubulin assembly and stabilize microtubules [44]. Hyperphosphorylated tau impairs its binding to tubulin; thereby induce instability of the cytoskeleton network. Clinical study showed that the level of hyperphosphorylated tau is significantly elevated in the brains of individuals affected from AD compared with the normal control. They can accumulate as intraneuronal tangles of paired helical filament, which is a pathological hallmark of AD. Activation of MAP kinases (JNK and ERK), and inactivation of protein phosphatase 2A (PP2A) to dephosphorylate p-Tau, have been proposed to play important roles during tau hyperphosphorylation [33,34,35]. In our cell model, p-ERK was markedly induced by the treatment of Hcy, however, was restored to the normal level by the addition of icariin in the cultured cortical neurons (Figure 5A,B). Icariin also suppressed the Hcy-induced p-JNK at a less striking level. The stress-activated kinase JNK molecule is one of the tau kinases and may involve in the pathogenic hyperphosphorylation of tau in AD [34]. Taken together, the neuroprotective effects of icariin might be, in part, linked to its ability to decrease the Hcy-induced p-tau, at the same time, to downregulated the phosphorylation of ERK. Besides modulating the cytoskeleton networks and the associated signaling molecules, icariin also showed to induce phosphorylation of AKT. Previous study indicated that icariin may prevent corticosterone-induced cell death via the activation of PI3-K/Akt pathway [36]. It is well known that Akt activation can trigger multiple cellular responses, including inhibition of apoptosis and stimulation of cell proliferation. However, the precise role of Akt in the neuronal protection of icariin in the present disease model requires further investigation.

The traditional wisdom has used anti-aging herbs, such as the medicinal herb *Epimedium brevicornum*, to prevent or slow down the deterioration of body functions during the aging process. To further investigate the mechanism underlined the protective effect of icariin, we applied the RT^2^ Profiler PCR Arrays specific for AD and neurotoxicity gene panels. From the AD Arrays, two genes, Adam9 and Apbb2 of the eight altered genes were differentially expressed between the Hcy and icariin/Hcy or icariin groups (Figure 7A,B). From the neurotoxicity arrays, we also identified two genes; Bdnf and Cd8b out of 18 altered genes were altered differentially between the Hcy vs. icariin or icariin plus Hcy groups (Figure 7C,D). The gene Adam9 is worthy mentioned. It was downregulated by 15 folds in the Hcy group compared to the control. Addition of icariin was able to bring back the Adam9 RNA level to the same level as the control. The Adam genes encode a large family of proteins possessing protease activities and many other functions [45]. The functions of Adam family are very diverting. Among them, ADAM9, ADAM10, and ADAM17 have been identified as α-secretase. Alpha-secretase, the gene product of Adam gene family, cleaves APP within the Aβ domain to release the AD-associated Aβ. It has been reported that Sirtuin type 1 (SIRT1), the longevity gene, increases the expression of ADAM10 gene encoding α-secretase which protects against accumulation of pathogenic Aβ peptide. Recent finding showed that icariin can suppress neuronal death induced by oxygen and glucose deprivation through the induction of SIRT1 [46]. SIRT1 was suggested to be a promising serum marker for early detection of AD in a recent report [47]. Interestingly, SIRT1 can be enhanced by the treatment of icariin in our study (unpublished data). The link between the neuroprotection of icariin and the ADAM family is of interest to be explored in the future.

## 4. Methods and Materials

### 4.1. Reagents

Icariin powder, ≥95% purity verified by HPLC, was obtained from Nanjing Ze Lang Medical Technology Co. (Nanjing, China). d,l-Homocysteine was purchased from Sigma-Aldrich, Inc. (St. Louis, MO, USA). Minimum essential medium (MEM), penicillin and streptomycin (P/S), fetal bovine serum (FBS), phosphate buffered saline pH = 7.4, (10×) (PBS), 0.25% trypsin-EDTA. Phosphatase inhibitors cocktail and poly-l-lysine hydrobromide were obtained from Sigma-Aldrich, Inc. Lactate dehydrogenase (LDH) cytotoxicity assay kit and protease inhibitor cocktail were from Roche Diagnostics (Mannheim, Germany). Rabbit polyclonal antibodies against p-Akt (Ser473), Akt, p-ERK, ERK, p-JNK (Thr183/Thr185), JNK, p-Tau (Ser400/Thr403/Ser404), Total Tau (Tau-5), mouse Tyr-tubulin and mouse anti-Ac-tubulin (Lys40) were purchased from Cell Signaling Technology (Beverly, MA, USA). Mouse anti-α-tubulin and phosphatase inhibitor cocktail were purchased from Sigma-Aldrich, Inc. Horseradish peroxidase (HRP)-conjugated goat anti-rabbit and goat anti-mouse antibodies were from DAKO (Glostrup, Denmark). Biomax X-ray film, Amersham enhanced chemiluminescence (ECL) detection kit and Amersham ECL plus detection kit were from GE Healthcare (New York, NY, USA). RNeasy mini kit, RT^2^ First Strand kit, RT^2^ Profiler PCR Array and 2× RT^2^ SYBR Green MasterMix was purchased from QIAGEN (Valencia, CA, USA).

### 4.2. Preparation of Primary Embryonic Culture of Rat Cortical Neurons and Hcy/Icariin Treatments

Primary embryonic culture of rat cortical neurons was established from embryonic day 18 Sprague-Dawley rat embryos via the method described previously. Cerebral cortices were micro-dissected from 18-day-old embryonic SD rats (The Laboratory Animal Unit, The University of Hong Kong) in 1× PBS with glucose supplement (18 mM). Cells were mechanically dissociated by repeated trituration with pipettes and then they were then seeded on 6-well plates or 15 mm glass cover slips pre-coated with poly-l-lysine (25 μg/mL) at a density of 8.0 × 10^5^ cells/well and 3.5 × 10^5^/coverslip, respectively. The cultures were kept in completed medium that consisted of minimum essential medium (MEM) supplemented with 5% heat inactivated fetal bovine serum, glucose (18 mM), l-glutamine (2 mM), insulin (5 μg/mL), progesterone (0.02 μM), putrescine (100 μM), selenium (30 ρM), penicillin (50 U/mL), streptomycin (50 μg/mL) and β-mercaptoethanol (25 μM). Neurons were maintained at 37 °C in a humidified 5% CO_2_ atmosphere. All experiments were performed with 7-day old cultures. Icariin was dissolved in DMSO to make a 100 mM stock stored at −40 °C as the stock solution. For experiment involved the use of Hcy, primary cultures of rat cortical neurons were kept in serum free MEM medium during treatment period. For combined drug treatment, primary cortical neurons were preincubated with different dosages of icariin for 1 h, then co-incubated with Hcy for the duration as indicated. After the incubation period, the cells were harvested for various biochemical tests.

### 4.3. LDH Cytotoxicity Assay

Neuronal cell injury in various treatment groups were detected by LDH assay. LDH, an enzyme that catalyzes the conversion of lactate to pyruvate is an important step in energy production in cell, is released from the cleaved cell membrane following cell damage insult. The assay was carried out using the LDH cytotoxicity detection kit. The release of LDH from the cell-free culture medium was determined by measuring the absorbance with a spectrophotometric plate reader at the wave length 492 nm according to the manufacturer’s manual. Results were expressed as the fold of control.

### 4.4. Protein Preparation for Western Blot Analysis

Equal amount of the protein sample were mixed with western-blot lysis buffer and protein was denatured at 95 °C for 5 min. Denatured protein sample was subjected to sodium dodecyl sulfate-polyacrylamide gel electrophoresis (SDS-PAGE) and separated in 10% SDS-PAGE gel. The resulting blots were hybridized separately with p-Akt (Ser473) (1:1000), Akt (1:1000), p-ERK (1:2000), ERK (1:2000), p-JNK (Thr183/Thr185) (1:1000), JNK (1:1000), p-tau (Ser400/Thr403/Ser404) (1:2000), tau, Ac-tubulin (1:40,000), Tyr-tubulin (1:20,000) and visualized using ECL-plus western-blotting detection kit. Optical density of the blots was measured with Image J software (National Institutes of Health, Bethesda, MD, USA).

### 4.5. Immunohistochemical (IHC) Staining 

The cytoskeleton integrity of primary cultures of cortical neurons was investigated through IHC staining. Glass cover slips were sterilized and coated with poly-l-lysine hydrobromide solution, then placed on the 35 mm dish. The cortical neurons were seeded on 35 mm dish at a density of 3.5 × 10^5^ cells/dish. At the end of the experiment, the cells-adhering cover slips were incubated with a solution of 0.1% Triton X-100 in PBS for 5–8 min, then incubated in 5% BSA 1 h for blocking non-specific binding. The primary antibody was diluted to desired dilution in 1× TBS and was added on the coverslip and incubated for 60 to 90 min. The cells were then incubated with secondary antibodies (diluted in 1× TBS) at room temperature for 60 min. The cover slips were mounted onto glass slides with anti-fade mounting solution. The processed cover slips were examined under a fluorescent microscope (Leica, Wetzlar, Germany).

### 4.6. RT^2^ Profiler PCR Arrays Analysis

Two RT^2^ Profiler PCR Array Kits focused on genes and pathways associated with AD and neurotoxicity was employed to investigate RNA profiles of neuronal cells treated with Hcy, icariin, or Hcy plus icariin. Total RNA of neuronal cultures was extracted and quantified using a RNeasy Mini kit according to the manufacturer’s instruction. The first strand of cDNA was synthesized based on the instruction of RT^2^ First Strand Kit. 1 μg total RNA was used for cDNA synthesis. Each RNA sample was incubated with 2 μL buffer GE and RNase-free water at 42 °C for 5 min, and then cooled on ice for 2 min. The reaction mixture which contained 5× Buffer BC3, Control P2, RE3 Reverse Transcriptase Mix and RNase-free water was then added into samples and incubated at 42 °C for 15 min. The reaction was terminated by heating at 95 °C for 5 min. The final cDNA product was used for the RT^2^ Profiler PCR Array using SYBR Green-based real-time PCR according to the manufacturer’s protocol. Each PCR Array contains 84 genes relevant to a specific pathway or disease state. All data were normalized to an average of five housekeeping genes β-2 microglobulin (B2m), Hypoxanthine-guanine phosphoribosyl transferase 1 (Hprt1), β-actin (Actb), Lactate dehydeogenase A (Ldha) and Ribosomal protein large P1 (Rplp1). Qiagen’s online web analysis tool was utilized to produce comparative heat maps, and fold change was obtained by determining the ratio of mRNA levels to control values using the ΔCt method (2^−ΔΔCt^). ΔΔCt = (Ct_treatment_target gene_ − Ct_treatment_reference gene_) − (Ct_control_target gene_ − Ct_control_reference gene_). Real-time PCR were carried out on ViiA™ 7 Real Time PCR System (Applied Biosytems, Grand Island, NY, USA).

### 4.7. Statistical Analysis 

Data were expressed as mean ± SE from at least 3 independent experiments. The significance of the differences among different groups was determined by one-way ANOVA, followed by Student Newman-Keules as post-hoc test, using Graph Prism Version 6.0 software (Graph Pad Software, Inc., San Diego, CA, USA). *p <* 0.05 were considered to be statistically significant.

## 5. Conclusions

**Figure 8:**
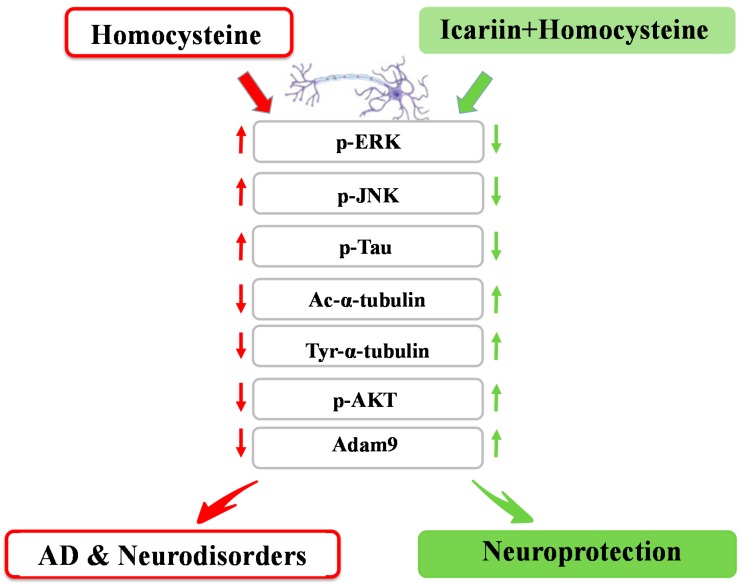
Signaling molecules and factors contributed to the neuroprotective effects of icariin in the primary embryonic cultures of rat cortical neurons. The red arrows indicate the Hcy-induced changes of the protein molecules, while the green arrows indicate the changes of protein molecules altered by the co-treatment of Hcy and icariin. ↑: upregulation; ↓: downregulation.

In this study, we present evidences that icariin from *E. brevicornum*, a commonly used anti-aging medicinal herb in traditional Chinese medicine, significantly attenuated the toxicity induced by Hcy. Using western blot analysis, IHC staining, and RT^2^ Profile PCR Arrays focused on genes associated with AD risk factors, we have revealed that icariin can effectively modulate the phosphorylation of signaling molecules, ERK, JNK, Tau and AKT to encounter the Hcy-induced damage of cytoskeleton networks and cell injury in the primary cultures of rat cortical neurons. The study also found that icariin markedly enhances a novel factor Adam9 whose expression may link to AD pathogenesis. The proposed underlying mechanism of the neuroprotective effect of icariin is summarized in Figure 8. Our findings suggest that icariin shows significant effects in the present in vitro disease model and may hence be an interesting lead molecule for further studies on AD or other neurodegenerative diseases.

## Figures and Tables

**Table 1:** RT^2^ Profiler PCR Array Gene Expression Analysis.

**Alzheimer’s Disease Array (Fold Change)**			
**Gene Name**	**Hcy**	**Icariin + Hcy**	**Icariin**
Gng11	2.61	1	1
Adam9	−15.66	1	1
Apbb2	−3.32	1	1
Serpina3c	−2.74	−3.08	−2.7
Bdnf	−2.6	1	1
Ins2	1	−2.35	1
Mpo	1	1	2.59
Plau	1	1	−2.15
**Neurotoxicity Array (Fold Change)**			
**Gene Name**	**Hcy**	**Icariin + Hcy**	**Icariin**
Angptl4	6.35	17.98	2.49
Arg1	4.49	4.92	2.08
Bdnf	5.83	6.57	2.57
Casp7	4.50	5.17	2.32
Cd8b	3.90	2.48	1
Tnfrsf25	8.42	8.12	10.20
Tph1	2.47	4.00	3.52
Notch4	−2.49	1	−2.71
Trpm4	−2.07	1	−2.45
Usp7	−2.11	1	−2.57
Clcnkb	1	2.09	1
Tp53	1	2.38	1
Cidea	1	1	−2.10
Cideb	1	1	−2.14
Ereg	1	1	−2.04
Heph	1	1	−2.51
Lta	1	1	−2.61
Nfkb1	1	1	−2.24

## Appendix

**Table A1:** Primer sequences.

Gene Name	Gene Full Name	Primer Sequence
**Adam9**	ADAM metallopeptidase domain 9	F: 5’-aattaactgcattagtgtaa-3’
		R: 5’-cctgcttctc tgtgtactaa-3’
**Apbb2**	Amyloid β (A4) precursor protein-binding, family B, member 2	F: 5’-aatgtgaagcgaggggtctt-3’
		R: 5’-gggcttctttagtgtagcta-3’
**BDNF**	Brain-derived neurotrophic factor	F: 5’-cgccactccgaccccgcccg-3’
		R: 5’-cctgcagccttccttcgtgt -3’
**Cd8b**	CD8b molecule	F: 5’-gactccttcatccctgctgg-3’
		R: 5’-tcctttggttgaagtccggg-3’
**Ldha**	Lactate dehydrogenase A	F: 5’-gggccattggcctctccgtg-3’
		R: 5’-ggatgcacccgcctaaggtt-3’
**Rplp1**	Ribosomal protein, large, P1	F: 5’-gcaccgtgccggcagtccac-3’
		R: 5’-gagggcggagtagatgcagg-3’
**β-actin**	Actin, beta	F: 5’-cagaaggagattactgccct-3’
		R: 5‘-acatctgctggaaggtggac-3’
